# Emotions and personality traits as high-level factors in visual attention: a review

**DOI:** 10.3389/fnhum.2012.00321

**Published:** 2012-11-29

**Authors:** Kai Kaspar, Peter König

**Affiliations:** ^1^Institute of Psychology, University of OsnabrückOsnabrück, Germany; ^2^Institute of Cognitive Science, University of OsnabrückOsnabrück, Germany; ^3^Department of Neurophysiology and Pathophysiology, University Medical Center Hamburg-EppendorfHamburg, Germany

**Keywords:** visual attention, high-level factors, emotion, personality, attention-emotion interactions, inter-individual differences

## Abstract

The visual sense has outstanding significance for human perception and behavior, and visual attention plays a central role in the processing of the sensory input. Thereby, multiple low- and high-level factors contribute to the guidance of attention. The present review focuses on two neglected high-level factors: emotion and personality. The review starts with an overview of different models of attention, providing a conceptual framework and illustrating the nature of low- and high-level factors in visual attention. Then, the ambiguous concept of emotion is described, and recommendations are made for the experimental practice. In the following, we present several studies showing the influence of emotion on overt attention, whereby the distinction between internally and externally located emotional impacts are emphasized. We also provide evidence showing that emotional stimuli influence perceptual processing outside of the focus of attention, whereby results in this field are mixed. Then, we present some detached studies showing the reversed causal effect: attention can also affect emotional responses. The final section on emotion–attention interactions addresses the interplay on the neuronal level, which has been neglected for a long time in neuroscience. In this context, several conceptual recommendations for future research are made. Finally, based on findings showing inter-individual differences in human sensitivity to emotional items, we introduce the wide range of time-independent personality traits that also influence attention, and in this context we try to raise awareness of the consideration of inter-individual differences in the field of neuroscience.

## Introduction

### Visual attention: models, definitions, and influential factors

Our view of the world to a large degree rests on the information we gather by the visual sense, and visual attention plays a central role in the processing of the sensory input. By directing one's attention to specific features in the environment, these features are processed in more depth than those environmental characteristics that are not the focus of attention (Chun et al., 2011). Several psychological and neuropsychological models of attention have been developed so far (e.g., Broadbent, 1958; Kahneman, 1973; Posner and Petersen, 1990; Mirsky, 1996; Coull, 1998; Corbetta and Shulman, 2012), but nonetheless a precise definition of attention is lacking. Currently, it is generally accepted that several sub-systems of attention can be differentiated.

Based on neuropsychological findings and single-cell recordings in monkeys, Posner and Petersen (1990); Petersen and Posner (2012) suggested three neuronal networks which are distinctive but collaborate to direct attention to specific locations in space. The posterior attentional (orienting) system prioritizes the sensory input by selecting a modality and location. The anterior attentional system is involved in detecting stimuli of current relevance by capturing awareness in a specific way and slowing detection of another potential target. This process is related to the limited capacity of the attention system and awareness, hence often labeled as focal attention. Finally, they postulated an alerting system being involved in preparing and sustaining alertness to process high-priority signals. This classical framework has recently been updated on the basis of new findings that deepen or expand the original networks (Petersen and Posner, 2012).

According to Coull (1998), one sub-system is responsible for directing attention to a stimulus (attentional orientation). A second sub-system mediates selective attention by giving focused attention to a particular stimulus while diminishing the attention to other stimuli (biased competition). A third sub-system provides divided attention between several stimuli even when they are spatially separated (Hahn and Kramer, 1998), and hence indicates that the current eye position does not necessarily correspond to the focus of attention. A fourth sub-system is additionally responsible for sustaining the attention to one or several (even non-contiguous) zones of the visual field (Müller et al., 2003).

Corbetta and Shulman (2012) found evidence for a dorsal frontoparietal network, including intraparietal sulcus, frontal eye field, and middle temporal complex, which was the primary network involved in selective attention to stimuli in the environment. Endogenous (i.e., voluntarily directed) shifts of attention activated the dorsal network, but it was co-activated with a second ventral frontoparietal network (right temporoparietal junction, right ventral frontal cortex, and insula), when unexpected but behaviorally relevant stimuli occurred at unattended locations. Hence, the ventral network was labeled as “stimulus-driven” (and was also often labeled as exogenous; for a detailed description of endogenous and exogenous shifts of attention, see Cormick, 1997).

Independent of whether these models are sufficient to explain all phenomena of attention, and independent of how the postulated sub-systems specifically interact on the neuronal and functional levels, the core aspect in all models is the bundling of cognitive resources to adequately process stimuli being important at a certain time. Correspondingly, attention often is metaphorically described as a moveable spotlight facilitating the stimulus inside its beam (Eriksen and Yeh, 1985; Posner and Petersen, 1990), whereby Desimone (1998) pointed out that the enhancing effect of attention on neuronal responses is perhaps better understood in terms of a competition among all of the stimuli in the visual field for control over behavior (biased competition hypothesis).

The central question that has motivated much research addresses factors influencing this attentional spotlight or the fate of sensory items during competitive interactions. Although not always explicitly mentioned, the visual system is the primary focus of models and experimental studies. Of course, due to the outstanding significance of the visual sense for human perception and behavior, visual attention adopts a key role in the selective choosing of input that, in turn, substantially constitutes the base for planning and controlling our interactions with the external world. Thereby, multiple factors contribute to the guidance of visual attention: on the one hand, the pure visual characteristics of the outside world, as well as the importance of objects for action control, substantially determine the course of the attention focus. On the other hand, visual attention is also heavily influenced by the internal state of the individual observer, constituted by a huge diversity of factors such as his current needs, the prevailing emotion, and motivational tendencies, as well as more time-independent personality traits. Hence, attention is not only stimulus-driven.

In the context of attention research, shifts of attention with saccadic eye movements (overt attention) as well as covert attention phenomena (attentional shifts without eye movements) have moved to the center of interest. However, as Corbetta and colleagues (1998, 761) pointed out, “the relationship of visuospatial attention and eye movements is controversial.” On the one hand, it has been known for a long time that humans can attend to objects out of their line of sight (James, 1890); i.e., it is possible to attend to several locations in the periphery while saccades are not allowed (e.g., Posner, 1980). On the other hand, a shift in overt attention seems to require a shift in covert attention (Hoffman and Subramaniam, 1995). Correspondingly, functional anatomical data showed that overt and covert shifts of attention not only are functionally related, but they also share parts of the same neuronal networks in the human brain (Corbetta et al., 1998; Beauchamp et al., 2000).

In the present review, we refer to both overt and covert visual attention and their potential interplay with specific higher cognitive functions. In this context, it must be noted that overt attention is not exclusively driven by external stimuli, just as processes of covert attention are not affected only by the inner state of the observer.

In order to clarify what we mean when talking about the impact of high-level cognitive mechanisms on overt or covert visual attention, we will briefly describe the two current main research fields dealing with overt attention, as they illustrate nicely the difference between low- and high-level factors in visual attention.

Many eye-tracking studies have addressed the impact of low-level image properties on eye movement guidance by comparing spatial image fixation probabilities. In this sense, the bottom-up selection of image locations involves fast stimulus-driven mechanisms such as a compulsory look to unique features (Treisman and Gelade, 1980) or at abrupt occurring stimuli (Yantis and Jonides, 1984). Indeed, sometimes we consciously recognize the strong impact of high salient objects, which seems to automatically attract our attention even if we try to defend ourselves against it. Hence, in literature, visual saliency is considered one of the main determinants of gaze control by first attracting our attention and then mediating viewing behavior (for a current review, see Schütz et al., 2011). Empirical studies suggest the significance of these bottom-up mechanisms on humans' attention: Several image features were shown to influence fixation behavior, such as spatial contrast (Reinagel and Zador, 1999), luminance and edges (Krieger et al., 2000), and color (Frey et al., 2007). In addition to task dependent information and spatial viewing biases, basic image features were found to make a significant and independent contribution to overt visual attention (Kollmorgen et al., 2010). Thereby, the impact of low-level image features was found to be stable across multiple viewings of complex visual scenes (Kaspar and König, 2011b).

Besides this impact of low-level image properties, high-level cognitive mechanisms are suggested as the second important influential factor on viewing behavior (Yarbus, 1967). Previous literature showed, for example, that a congruency between a stimulus and the content of the working memory can early and involuntarily attract attention (Soto et al., 2005). Also, the current behavioral mode seems to affect the allocation of attention, since drivers and passengers in a virtual environment were found to differ in their sensitivity to changes in the environment (Wallis and Bülthoff, 2000). Moreover, in the context of eye-tracking studies, sometimes the effects of the current task on viewing behavior (e.g., browsing mode versus search mode on web pages) are explicitly considered (Triesch et al., 2003; Nelson et al., 2004; Rothkopf et al., 2007; Betz et al., 2010; Hamborg et al., 2012), as are effects of age on attention (Mather and Carstensen, 2003; Acik et al., 2010). However, beyond these factors, there are further substantial mechanisms affecting attention control. They range from the current emotional state of the observer and his motivational state to time-invariant personality traits. So far, few studies have addressed these issues, although even saliency models explicitly point out that high-level cognitive processes can significantly affect the formation of a current saliency map. In the following, we give a selective review of studies which focused on these high-level mechanisms in the context of overt as well as covert attention processes.

Although a considerable amount of research is devoted to visual attention, a commonly used definition or model of attention is lacking. Several models have been proposed by now, each highlighting important aspects of attention. All in all, attentional processes seem to be more complex, as suggested by the simple spotlight metaphor. The distinction between overt and covert attention shows that shifts in attention are not necessarily paralleled by shifts in eye movements. In this context, overt attention elegantly allows the study of the impact of high- vs. low-level factors in visual attention, as eye movements and their potential correlation with image properties can be controlled while high-level factors are manipulated (and vice versa).

While the impact of low-level images on overt attention has been extensively investigated in the last two decades, the impact of high-level factors on attention (overt and covert) has been neglected. The present review intends to give an overview of various, partially heterogeneous, studies on two widely neglected high-level factors in visual attention (emotion and personality) in order to give starting points for future research as well as point out important methodological aspects that have be addressed appropriately.

### The ambiguous concept of emotion

Is there a point in time when we are completely free of any emotional impact? Probably not! Of course, often we are not aware of the specific emotion that influences our current thinking and behavior. In contrast to other cognitive or physical processes such as motivation, a specific intention or a present pain might not be in the foreground of conscious perception, but might modulate our mental state. Yet, specific emotions do correlate with the arousal level and hence with the degree to which we are sleepy or focused in an attentional sense. Thereby, it seems fruitful to distinguish between the two constructs of emotion and arousal. According to Kensinger (2004, 241), “a widely-accepted framework proposes that affective experiences are best characterized in a two-dimensional space.” In one dimension, valence ranges from highly negative to highly positive, and in a second orthogonal dimension, arousal ranges from calming to exciting. In several previous studies, it was neither obvious nor explicitly mentioned what dimension was addressed by the experimental manipulation, which hinders comparison or integration with further research. With a view to the current literature, the common labeling appears to be “emotional valence” and “emotional arousal,” respectively. However, sometimes the term “affect” seems to be used instead of valence (e.g., Isen et al., 1987). This distinction is important, as changes in both arousal and valence do not necessarily lead to identical neuronal activation, as found by Lane et al. (1999). They showed that extrastriate visual cortical and anterior temporal areas were independently activated by emotional arousal and valence. On the other side, emotional arousal and valence were also found to interact under certain circumstances: Adolphs et al. (1999) investigated the recognition of emotional arousal and valence in a subject with complete bilateral damage restricted to the amygdala. Recognition of emotional valence was normal, but recognition of emotional arousal was impaired for unpleasant facial expressions, words, and sentences. Additionally, memory can be enhanced for negative or positive stimuli which do not evoke arousal (Kensinger and Corkin, 2003).

However, this simple orthogonal relationship between valence and arousal indeed facilitates theory-building and experimental operationalizations, but there is also evidence for a more intermingled relationship between both constructs. Several theorists postulated valence or arousal as fundamental for emotional experience (e.g., Ortony et al., 1988; Lazarus, 1991); others incorporate both aspects (e.g., Lang, 1994). Furthermore, some authors, such as Eysenck (1981), declared that emotion results from arousal. In contrast, others view arousal as a result of emotion (Gray, 1981).

Moreover, Barrett (1998) showed that people differ in how they label their own emotional states. People high in valence focus but low in arousal focus refer to a dimensional model when labeling their emotional states. In contrast, people high in arousal focus but low in valence focus prefer a discrete emotion model.

Finally, Derryberry and Rothbart (1988) pointed out that the unitary nature of both constructs can be questioned. They determined that at almost every level of physiological analysis (cortical, autonomic, and endocrine), arousal was revealed to be a multidimensional set of processes. Similarly, the limbic system, with its numerous circuits, allows a variety of emotional, perhaps separable, systems controlling different emotions such as fear, pleasure, or frustration. In this context, however, neuroscience shows that it could be an obstacle if emotional experiences were assigned to discrete categories such as fear, anger, or happiness. On the neuronal level, the search for co-occurring sets of neuronal features that differentiate between such categories was not successful (Barrett, 2006). On the other hand, Talarico et al. (2009, 382) emphasized that “although dimensional accounts of emotion are informative, the influence of discrete emotions should not be underestimated.”

Consequently, as with attention, no common sense seems to exist regarding a definition and theoretical framework of emotion. However, some practical recommendations can be made: One should not only define the type of emotional dimension (arousal and valence) that is manipulated or investigated, but also specify the kind of emotion or affect as clearly as possible. The term *mood* is sometimes used to describe the inner state of participants (as shown below), but it should be avoided against this background, because it is too general and does not allow a clear categorization of the observer's current emotional state. Clear labeling will facilitate the interpretation of data, and hence the comparison with other studies will become easier and more unambiguous. Advantages and disadvantages of a dimensional versus categorical framework for emotional experiences have to be assessed (see Section “Interaction Between Emotional and Attentional Processes on the Neuronal Level”).

## The impact of emotion on attention

In studies dealing with the potential impact of emotion on other cognitive processes, a neutral emotional state is commonly used as a baseline and then contrasted with salient emotional states such as an exalted joy or strong fear. These more pronounced emotional states are in general characterized by higher valence or arousal. For example, the impact of emotional arousal on the sensitivity of several cognitive functions has been investigated in multiple studies: recall of peripheral details of an event is improved when being in a positive emotional state in contrast to a neutral state (Talarico et al., 2009). Furthermore, positive affect, in contrast to a negative effect, can boost performance in creative solving tasks (Isen et al., 1987) and also enhances performance in solving resource dilemmas (Knapp and Clark, 1991). Given these effects on memory and higher cognitive functions, it is only a small step to the question of to what degree the emotional state of an observer influences visual attention and perception.

Based on this central question, Wadlinger and Isaacowitz (2006) investigated by means of tracking eye movements how a specific mood affects the attentional preference to emotional stimuli. Individuals induced into positive mood, in contrast to a neutral mood, fixated more on peripheral stimuli than did control participants and displayed a broadened attention distribution. However, this only held true for high-valence positive stimuli, while the arousal ratings of all images were similar. Participants under induced positive mood additionally made more frequent saccades for slides of neutral and positive valence. Hence, they found a significant effect of priming a positive mood.

According to Frederickson (2000), the fundamental function of positive emotions is to broaden the momentary thought-action repertoire of an individual, in contrast to negative emotions that narrow this repertoire toward specific actions in order to serve capacities for survival-related actions. However, this broader focus of attention is not limited to positive stimuli in the environment. Several studies have shown that persons being in a positive emotional state attend to negative information when it could be of future advantage to them. For example, Reed and Aspinwall (1998) provided evidence that when recalling positive experiences of the past, the biased processing of self-relevant health-risk information was reduced. This finding is not compatible with an alternative explanation of the broader focus of attention to positive stimuli evoked by a positive mood: Wadlinger and Isaacowitz (2006) also considered the possibility that the broader distributed attention to positive stimuli may act to maintain the current positive emotional state.

Here, a general distinction is important when talking about the impact of emotion on attentional processes: on the one hand, many studies focus on the impact of emotional stimulus properties on the attentional focus and the perception of those emotional stimuli. On the other hand, one can also be interested in the impact of the observer's current emotional state on the course of attention, irrespective of whether the observed stimulus itself is emotion-laden. In the first case, attention is directly driven by the emotional features of the stimulus and hence is potentially related to so-called bottom-up processes in the visual hierarchy. In the second case, the emotional component playing a role in attentional shifts is located within the observer and hence affects attention processes on subsequent stimuli in a more top-down manner. In the following, we will speak of an externally located influence when referring to the impact of emotion-laden stimuli, and we will speak of an internally located impact when referring to the observer's current emotional state.

The distinction between internally and externally located emotional influences facilitates the evaluation of attentional processes affected by emotion. However, this distinction is not without problems. The internally located impact of emotion can be undoubtedly assumed as far as the emotional state of an observer precedes and is therefore not dependent on the stimulus. The stimulus-driven way can be assumed for certain if the observer was in a neutral emotional state when confronted with the stimulus. Unfortunately, both cases rarely occur in this pure form. Thereby, investigations on the impact of externally located emotional properties on attention and perception can be handled: given an adequate experimental procedure controlling for a systematic covariance between dependent and confounding variables, inter-individual differences in the current emotional state are balanced out and hence effects between experimental conditions can be derived from the experimental manipulation (i.e., the visual input). For example, in order to investigate the impact of positive vs. negative priming on viewing behavior under natural conditions, potential inter-individual differences in the current emotional state have to be equalized by randomization of subjects to treatments (given a high number of subjects). Otherwise, differences between the priming conditions regarding viewing behavior could be derived from systematic differences between subjects' pre-experimental emotional states, but not from the treatment.

In contrast, the internally located impact of emotion on perception is more difficult to adequately manipulate and control. One critical parameter is the time interval between the experimental induction of a certain emotional state in the observer and the observation process. This interval moderates the strength of the emotional effect on attention. Emotional processes and changes are linked to the hormone system working slower than neural processes. Hence, the strength of an emotion induction reaches its maximum with a temporal delay, and the level of arousal is variable over time. Therefore, some researchers decided to implement an emotional manipulation that works continuously during the whole experimental session to maintain the emotional state. For example, Hirschberger et al. (2010) introduced permanent subliminal emotional priming in order to maintain a context of negative valence while recording subjects' fixation behavior on a four-image cluster.

A further difficulty for investigations on the internally located impact of emotions on attention and perception is the fact that the visual stimuli observed are not emotionally neutral in most cases. Many pictures of the International Affective Picture System (IAPS) Lang et al., 2005) depict scenes and objects comparable to those used as target images in many studies, but valence and arousal ratings for the IAPS images show that most pictures are not (and should not) be perceived as neutral. Consequently, it cannot be ruled out that an induced emotional state, i.e., the internally located impact of emotion, interacts with the externally located impact of emotional stimuli. Finally, the observation of specific emotion-laden stimuli in a sequence can result in a more and more internally driven impact of emotion, although the researcher is exclusively interested in the impact of image properties on perception processes. Importantly, in addition to the division of an internally and externally located impact of emotion on attention, researchers sometimes use stimuli showing the emotion of somebody else (e.g., Vermeulen et al., 2009; see below), which is often taken as shorthand for an emotionally positive or negative stimulus. It might indeed induce the same emotion, yet it is a very specific choice. These interactions and complexities show that a clear distinction between emotional bottom-up and top-down influences on perception is a difficult venture, and we have to be careful when inferring from study results the exact entity of the emotional impact on perception.

The interaction of internally and externally located emotional effects is demonstrated nicely in recent eye-tracking studies (Kaspar et al., accepted): Sets of complex scenes with high positive or negative valence as well as neutral images were used as supraliminal emotional primes to build corresponding emotional contexts. Nature scenes served as neutral target stimuli and were shown embedded in the three sets of primes. Subjects did not differentially scan the primes, but viewing behavior on neutral target images was significantly affected by the valence of the emotional context. Consequently, no simple transfer effects from the primes to the targets occurred on the level of eye movements. Rather, the results suggest that viewing behavior was indirectly influenced by the effect of prime valence on the inner state of the observer. Reducing the intensity of emotional priming and embedding single positive and negative primes in a train of neutral images belonging to different categories, negative primes were scanned more actively than their positive counterparts. This applies to fixations of shorter duration, longer saccades, and a more spread-out fixation distribution. Again, the signature of eye movements on primes was not transferred to neutral target images, but viewing behavior on targets was influenced by the valence of the preceding prime. These results illustrate the complex interaction between internally and externally located effects of emotion on attention.

Hirschberger et al. (2010) were interested in whether even unconscious negative priming influences the observer's viewing behavior when confronted with emotionally negative target images. In the experimental condition, a subliminally presented negative prime (the word *death*) was present across the whole duration of an eye-tracking session. In the control group, a neutral prime was presented and without any specific emotional manipulation. In their first experiment, subjects consecutively observed several four-image patterns depicting three neutral images and one picture showing physical injury. Participants were asked to recognize the images at the end of each trial in order to enhance their motivation to carefully observe the images. The results showed that the negative prime decreased gaze duration toward pictures of physical injury, but did not have an effect on gaze duration toward neutral images. Hence, participants in the negative priming condition spent less time looking at the pictures overall in contrast to subjects in the neutral priming condition. The authors concluded that the results are a signature of motivated unconscious attention, since subliminally presented negative primes evoked a strong influence on the amount of attention that is directed at meaningful stimuli.

In addition to the impact of emotion on eye movements, it is only natural to ask for target detection in a search task. Accordingly, researchers also have already investigated whether perceived emotions affect target detection. Vermeulen et al. (2009) used the attentional blink paradigm to demonstrate that processing fearful versus disgusted faces has different effects on attentional processes. In the attentional blink paradigm, a pair of targets is presented consecutively within a small time window. Thereby, the second target is normally missed when presented 200–500 ms after the offset of the first target (e.g., Raymond et al., 1992). Vermeulen and colleagues presented either a fearful or disgusted face as primes followed by a rapid sequence of distractors consisting of random strings of symbols and digits. Two target words of the same length were embedded into this sequence. Subjects had to detect both words, but results showed that processing fearful faces (which were previously assumed to enhance the allocation of attentional resources) impaired the detection of the second target word to a greater extent than did the processing of disgusted faces (which were expected to diminish the allocation of attentional resources). Obviously, the kind of emotion that is perceived plays an important role and has to be considered when investigating emotional effects on attention and perception, because the induction of a negative emotion leads to a reduced spread of attention not only in space, but also in time. However, it must be pointed out that it is unclear to what extent the short presentation of one second of emotional faces in this study affected the emotional-state of the observer. Moreover, it is important to note that the observation of emotional faces does not have the same effect as the observation of scenes depicting emotional content. Hariri et al. (2002) compared the response of the amygdala to fearful and threatening faces with the response to corresponding complex scenes. They found a significantly greater amygdala response to faces in contrast to scenes. When we observe emotional faces, we look at another person, but we do not have direct sensory access to what is affecting the other person. The amygdala obviously responds more strongly to such indirectly mediated emotions. However, especially for studies on overt attention, not only the affective impact of images should be considered, but also the image characteristics which revealed a very strong impact on the attention focus (see, e.g., Kaspar and König, 2011a,b). In general, many studies use human faces to induce different emotion conditions. Perhaps this is due to the fact that facial expressions are relatively easy to interpret for an observer, and hence specific emotions are recognized faster and without ambiguity. Interestingly, it seems that the emotion expressed by a face is more important than that face's corresponding physical appearance. Lamy et al. (2008) presented the first report of emotional priming of pop-outs. In the basic pop-out task, a target differs in a specific feature from multiple simultaneously presented distractors (Maljkovic and Nakayama, 1994). Remarkably, when in subsequent trials the identity of the target is repeated, detection is accelerated. In the study of Lamy and colleagues, participants repeatedly saw arrays of four faces and had to detect a discrepant target face that differed in the expressed emotion. In line with the classic pop-out effect, the detection of the target face was faster when the same emotional target face was presented on successive trials. However, this effect only occurred for angry and for happy faces embedded in an array of neutral faces, but neutral target faces displayed in parallel with emotional faces were not detected faster. Importantly, this effect completely disappeared in trials in which all faces (target and distractors) were inverted instead of upright. This result indicates that the faces' emotional category, rather than their physical properties, was the determinant for this pop-out effect.

To conclude, the current emotional state of a person does not only affect recall performance, performance in creative solving tasks, or in solving resource dilemmas. Moreover, emotion also was revealed to be a significant factor influencing humans' visual attention, but the distinction between an internally and an externally located impact of emotion seems necessary in this context. Both externally located emotion-laden stimuli and the internally located current emotional state of an observer affect overt attention. Thereby, the kind of emotional stimuli (faces vs. scenes) is relevant, and the study design as the manipulation of emotions is a challenging task.

## Perception of emotion outside the focus of attention

If no eye movements are recorded, as in the study of Lamy et al. (2008), we will not be able to assess the degree to which overt and covert attentional processes contribute to observed effects. In fact, several studies have shown that covert attention also plays a crucial role in the context of emotion's impact on attention and perception processes.

To further disentangle the interaction of emotional stimuli and attention, it is a necessary step to present primes outside of the focus of attention. Phelps et al. (2006) investigated the influence emotions have on the interaction of covert attention and perception. Subjects had to perform a two-alternative forced choice orientation discrimination task. One or four faces were presented in the periphery, followed, after a short delay, by four Gabor stimuli; one of these served as the target. Presentation times of all stimuli were short to exclude eye movements and ensure that covert attention was measured. They found that subjects were most sensitive to stimulus contrasts when they previously observed one face in the periphery that had a fearful expression in contrast to a neutral expression. Presently, it cannot be excluded that fearful faces elicited an unspecific arousal; Phelps and colleagues argue, however, that this effect was derived from the specific negative valence of the primes. This implies that emotion facilitates early visual processing. In other words, an internally located impact of emotion can be induced without directing one's attention to emotion-laden stimuli.

In more natural situations with complex arrangements of objects, the question arises whether humans extract the affective content without fixation. Calvo and Avero (2008) used pleasant and unpleasant complex images as primes that significantly differed in valence and arousal. The primes were shortly presented in parafoveal locations, followed by a blank and then by a foveal probe stimulus being either congruent or incongruent in emotional valence with the preceding prime. Participants had to promptly signal whether this probe was positive or negative by pressing a key. The results showed that congruent prime–probe pairs were detected faster than incongruent pairs when the prime–probe stimulus onset asynchrony was 300 ms and the prime appeared in the left visual field. This priming effect occurred regardless of differences between prime and probe in physical appearance and semantic category, since the assignment of primes and probes was random and both depicted scenes involving either people or animals. No effect was found when the prime was presented in the right visual field or when the prime-probe asynchrony was 800 ms. Hence, this study illustrates an interaction of externally and internally located influences of emotion, because the stimuli themselves were emotion-laden, but the priming also induced some emotional processing in the observers and affected their reactions to the probes. The authors concluded that this result indicates that even complex emotional stimuli can be assessed early by covert attention processes, and that a dominance of the right hemisphere is involved.

Other studies scrutinized whether emotional stimuli embedded in a bulk of distractors preferentially attract attention. That is, do emotion-laden stimuli pop out in the visual field and attract attention faster than neutral stimuli do?

In a series of experiments by Eastwood et al. (2001), participants had to detect either a unique positive or negative target face while observing displays of several schematic faces. All distractors were neutral faces, whereby the display positions of faces and the number of distractors were randomly varied in each trial. Subjects had to press a key once the target face was localized while maintaining high accuracy. Once the key was pressed, all faces were covered with gray squares and subjects had to indicate the square that corresponded to the location of the target face. Negative faces were detected faster than positive faces, and hence the results suggest a differential guidance of focal attention. To rule out that this effect was derived from the difference in physical image properties between positive and negative faces instead from their valence differences, Eastwood et al. conducted a further control experiment in which all faces were rotated by 180°. This change in face orientation led to a disappearance of differences in reaction time between the positive and negative face condition. Hence, the higher effectiveness with which faces showing negative expressions guided focal attention actually was based on the specific valence, i.e., a holistic processing of the face configuration, and not on local low-level image properties. This study suggests that externally located emotional influences preferentially attract humans' attention, whereby the valence of the stimulus seems to play a crucial role.

However, results are mixed in this regard. When embedded in an array of neutral faces, subjects locate happy or surprised faces faster and with higher accuracy than they do angry or fearful faces (Calvo and Nummenmaa, 2008). Moreover, a second experiment revealed that subjects fixated on and localized happy faces more often first and earlier than they did any other face. Faces were compared on several physical, i.e., basic image, properties such as luminance, contrast density, and global energy, as well as color and texture similarity. This analysis revealed that happy faces are more visually salient than the other emotional faces. In a third experiment, the procedure was similar to the first experiment, but all faces were inverted instead of having an upright orientation. This led to a disruption of the holistic configuration but left local facial properties unaltered. The setup with inverted faces replicated the pattern of differences in search time and accuracy found before. Furthermore, the search time and accuracy were worse for inverted faces in general, but the inversion only had a significant influence on the detection of sad, fearful, and angry expressions. Happy, surprised, and disgusted faces were not significantly affected. Hence, these results are consistent with a featural, rather than a configural, explanation. This advantage for happy face detection was attributed to the perception of single features rather than to the emotional meaning of faces. In further studies, Calvo and Nummenmaa (2008) were able to identify which facial features are responsible for this advantage. Their results suggested that the smiling mouth is responsible for facilitating the initial orientation to happy faces.

In contrast, a study by Fox et al. (2000) observed that people detected a discrepant face in an array of several faces faster when it displayed angry or sad, rather than happy, expressions. Importantly, this effect disappeared when the faces were inverted or when the mouth was presented in isolation. Interestingly, in a follow-up study, Fox and Damjanovic (2006) showed that the eye region alone can produce this threat of superiority effect in search tasks. The degree of the superiority effect did not increase with whole-face stimuli in contrast to eye-only stimuli. Therefore, the authors concluded that eye configurations are a key signal of threat, whereas Calvo and Nummenmaa (2008) found the smiling mouth to be responsible for the superiority effect of happy faces. Obviously, much more research is necessary to uncover the principles of the superiority effects of emotional facial expressions.

Given that emotional faces attract attention more than neutral faces do, researchers have begun to locate brain regions linked to this perceptual phenomenon. Vuilleumier (2002) pointed out that visual responses in the fusiform cortex were enhanced for emotional faces, consistent with their greater perceptual saliency. He also referred to data from event-related evoked potentials and neurophysiology that suggested rapid parallel processing of emotional information of sensory inputs. Moreover, processing of emotional information led to a more detailed perceptual analysis of the sensory inputs and hence biased competition for attention toward the representation of emotionally salient stimuli.

However, it is controversial to what extent emotion-laden stimuli are processed without attention. In general, the model of attention proposed by Desimone and Duncan (1995) suggests a competition of stimuli for neural resources. Spatially directed attention can bias this competition among multiple stimuli and enhance the impact of the attended stimulus. For example, Reynolds et al. (1999) have investigated the impact of focused attention on neurons in macaque areas V2 and V4 (see also Reynolds and Heeger, 2009). Presenting an additional stimulus in the receptive field reduced the neuronal response to the primary stimulus. However, when the monkey's attention was directed to one stimulus, the suppressive impact of the second stimulus was reduced. The reduction of the suppressive influence of such distractors has also been shown in humans by fMRI studies (Kastner et al., 1998). Hence, it appears that processing outside the overt focus of attention is weakened or even eliminated under certain conditions (Pessoa and Ungerleider, 2004).

In contrast to these findings, several studies suggest that the role of attention is different with respect to the processing of emotion-laden stimuli. For example, Whalen et al. (1998) investigated whether the amygdala is activated when human subjects observe masked emotional stimuli. They subliminally presented fearful or happy faces, followed immediately by a neutral face serving as a backward mask. Although subjects reported conscious perception of neutral faces only, the BOLD fMRI signal in the amygdala was higher during the observation of masked fearful faces in contrast to masked happy faces. Pessoa and Ungerleider (2004) interpret these data as showing that the amygdala is specialized for the fast detection of emotionally relevant stimuli in the environment, whereby this detection process can occur even without attention. In contrast to this view, they found that such a differential response of the amygdala to emotional faces significantly depends on the extent to which attentional resources are available for face processing: Participants were confronted with a foveally presented fearful, happy, or neutral face and additional bars located in the left and right periphery (Pessoa et al., 2002). Subjects always fixated on the face during the short presentation. In one condition, they had to direct their attention to the face in order to indicate whether the face was male or female. In a second condition, attention was directed to the bars in order to indicate whether they were of the same orientation. This demanding bar orientation task was intended to attract all attentional resources. The results showed that attended faces in contrast to unattended faces elicited greater activations bilaterally in the amygdala for all facial expressions. The authors concluded that contrary to the prevailing view, emotional faces are not immune to the effect of attention. In summary, evidence for an attention-free processing of emotional items is mixed, and future research is mandatory.

## The reversed way: attention can affect emotional responses

Given the extensive evidence suggesting that externally located emotional stimuli as well as internally located affective states determine how visual attention is allocated, the question arises whether this causal effect is also working in the reverse direction. In the following, we discuss an interesting way in which attention can influence emotional perception. In the study by Raymond et al. (2003), participants initially saw two schematic faces showing the same affective expression (positive, negative, or neutral). The faces were placed on the left and on the right of a fixation cross. Both faces were replaced by two complex but meaningless visual patterns differing in structure. At the beginning of each experimental block, one of these two visual patterns was designated as the target and participants were told to locate the target pattern as fast as possible by pressing a corresponding key after stimulus onset. After participants had responded, one of the two visual patterns previously presented occurred in isolation. Now, subjects had to judge whether this meaningless visual pattern appeared to be cheery or dreary. The results showed that the previously attended visual target pattern was judged more positively than the ignored distractor, which in turn was judged more negatively than a novel visual pattern. Hence, not the act of attending, but the active ignoring of the distractor led to its affective devaluation. Obviously, attentional inhibition of task-irrelevant stimuli (distractors) leads to a devaluation when the previously ignored stimulus is encountered again (Fenske and Raymond, 2006).

In further studies, Raymond et al. (2005) investigated evaluations of Mondrian-like patterns. Participants had to search for a target among a varying number of distractors that appeared simultaneously. Immediately afterwards, participants rated the affective tone of either a prior target or a prior distractor. Relative to the ratings of the prior targets, the magnitude of the distractor devaluation was greater when the distractors were presented at their original screen locations during evaluation, in contrast to the case when the distractors appeared at different locations. Moreover, distractor devaluation was generally enhanced when the distractor was located near the target, and it was attenuated when the target was further away.

As shown by Mounts (2000), the potential of distractors to interfere with the current performance is greater when a distractor is near the task-relevant target location. Consequently, to prevent reduced performance, the inhibition of distractors presumably increases with higher proximity to the target. In this context, Fenske and Raymond (2006) interpret the finding that the proximity of a distractor to a target strengthens the devaluation of the distractor in terms of a positive correlation between the amount of inhibition and the amount of distractor devaluation.

From our point of view, the attention's influence on emotion formation suggested by these findings should motivate further research in this direction. If bottom-driven attention truly constituted emotional ratings or even the emotional state of the observer, it would and should be a high priority to consider this causal effect when investigating subjective data as well as psychophysiological data.

## Interaction between emotional and attentional processes on the neuronal level

Emotional as well as attentional processes have been attributed to dynamic processes in large-scale networks. With respect to the neuronal level, the central question is, where do these two processes interact? Numerous authors have addressed these processes individually (see Bisley, 2011; Lindquist et al., 2012 for reviews on the neuronal basis of attention and emotion respectively). The interplay between them has been neglected for a long time, as pointed out by several authors (e.g., Carretié et al., 2001; Taylor and Fragopanagos, 2005), but now has moved into the focus of current research.

One functional pathway can be outlined against the background of the original model of attention postulated by Posner and Petersen (1990). The posterior attentional system involved in orienting processes receives strong innervation by pathways of the norepinephrine system arising in the locus ceruleus. The norepinephrine innervation is primarily present in the posterior parietal cortex, the superior colliculus, and the thamalic pulvinar. Hence, it seems to play a crucial role in maintaining alertness. The amygdala, in contrast, is a key structure of the emotional system and has a key role in regulating arousal (Williams et al., 2005). Furthermore, it is functionally related to both above-mentioned dimensions of emotion, namely valence and arousal. For an interaction between valence and arousal in the amygdala, see Garavan et al. (2001). Sterpenich and colleagues (2006) found the locus ceruleus to respond during correct recognition of neutral events encoded in an emotional context, whereby this response linearly depends on the autonomic arousal. Furthermore, the LC and the amygdala are functionally more connected during correct face recognition in emotional contexts than in neutral contexts. Obviously, the amygdala modulates emotional memories not only by affecting hippocampal activity, but also indirectly by influencing the central arousal. Perhaps the interplay of the locus ceruleus and the amygdala is a key link for the attention-emotion interaction.

With respect to the ventral frontoparietal network in the model of Corbetta and Shulman (2012), a link to emotion was also recently found: the ventral orienting network includes the inferior frontal cortices, which probably process emotional communicative information based on visual or auditory input (Nakamura et al., 1999). In line with this, Lim et al. (2009) combined the attentional blink paradigm with aversive conditioning and found evidence that the impact of the amygdala on visual cortical responses was partially mediated by the frontoparietal attention network. This result suggests that the fate of a visual stimulus during competitive interactions is determined by its affective significance, affectively significant stimuli being favored.

A further hint for emotion–attention network interactions resulted from a visual oddball task combined with fMRI measurement (Fichtenholtz et al., 2004): Participants had to detect rare target stimuli embedded sequentially in a stream of standard stimuli. Fichtenholtz et al. chose squares as the standard stimulus, occurring in 84.4% of trials. Circles, neutral complex scenes, or aversive complex scenes occurred at low rates. One group of subjects counted circle images; a second group counted aversive stimuli. The experiment revealed a higher activation in the amygdala and ventral frontotemporal cortices for emotional rather than for neutral scenes. Furthermore, a main effect of the attention focus in dorsal frontoparietal cortices was observed in terms of a greater activity for task-relevant target images irrespective of emotional content. Importantly, an interaction between the emotional and attentional focus was exclusively located in the anterior cingulated gyrus. When circles were task-relevant, the response of the anterior cingulated gyrus was equal to the response to circle targets and emotional scenes serving as distractors in this group. However, the activity of anterior cingulated gyrus increased when the emotional scenes were task-relevant, but decreased for distracting circles. These results highlight the integrative role of the anterior cingulated gyrus in the context of emotion–attention interactions.

Finally, Lane et al. (1999) used the PET technique to measure brain activity while male participants observed emotion-laden IAPS images controlled for valence and arousal. Participants simultaneously performed a distractor task in which they had to respond to auditory tones during image observation. A lowly and a highly distracting task were employed to manipulate the extent to which attention was absorbed by the secondary task. The results showed that the activation of the extrastriate visual cortices and the anterior temporal areas was independent of the emotional valence and arousal of stimuli and independent of the attentional resources. In contrast, there was an overlap in activation patterns associated with emotional arousal, emotional valence, and attention in extrastriate visual cortex centered in the right Brodmann areas 18 and 38 (the right anterior temporal cortex). These results suggest a common modulation of visual processing by emotion and attention at very early as well as late stages of cortical analysis.

Some authors devoted to this interaction provide first literature reviews (e.g., Compton, 2003; Schupp et al., 2006) and develop a model of emotion–attention interaction (Taylor and Fragopanagos, 2005). Besides these important works, future research and theory formation would benefit from considering several factors that potentially affect results:

1. First, it seems useful to envision the analogy between the general effects of attention and emotion on the behavioral level (c.f. Lane et al., 1999). As outlined above, in terms of the spotlight metaphor, attention enhances the processing of specific target stimuli by reducing the cognitive resources allocated to alternative stimuli. A similar effect can be observed regarding emotion: as Kensinger (2004) pointed out, emotional stimuli are more likely to be processed when attentional resources are limited, suggesting a prioritized processing of emotional information. This conclusion is supported, for example, by the findings of Anderson and Phelps (2001). They used the attentional blink design and found the second target was missed fewer times when it was a word of aversive content in contrast to a neutral word. Moreover, in comparison to neutral scenes, images depicting unpleasant or pleasant content are associated with increased early posterior negativity, late positive potential, and sustained positive slow wave (Schupp et al., 2006). The observation of emotion-laden stimuli also enhances the activity in visual brain regions that are associated with object recognition, such as inferotemporal cortices and the fusiform (Sabatinelli et al., 2004). Interestingly, patients with damage in the amygdala do not show a modulation of attention by emotional arousal (Kensinger, 2004). Correspondingly, Compton (2003) emphasizes a two-stage process in the context of emotion–attention interaction: in the first stage, a subcortical circuit involving the amygdala evaluates pre-attentively the emotional significance of a stimulus. In the second stage, stimuli tagged as emotionally significant are prioritized in the competition for access to selective attention.
  To conclude, for research on effects of selective attention and biased competition, it appears fruitful to explicitly consider the modulating effect of arousal. Effects found in non-arousing situations change when emotions come into play. Correspondingly, Mather and Sutherland (2011) provide evidence that emotional arousal amplifies salience based on perceptual contrast (bottom-up) as well as the top-down competitive advantage of high- vs. low-priority stimuli. Arousal stimulates the amygdala which, in turn, modulates sensory processing, frontoparietal attentional networks, and memory consolidation. Accordingly, the authors labeled the impact of emotion as “arousal-biased competition” in perception. Consequently, the usage of emotion-laden stimuli, for example, will change the neuronal areas of significant activity compared to emotionally neutral conditions.
2. As outlined above, the distinction between arousal and valence is useful in the context of emotions. Hence the question arises whether emotional valence also affects biased competition. In short, previous studies found arousal more important than valence in biased competition, but positive and negative valence have opposite effects on selective attention, and they additionally interact with the arousal level (for details, see Mather and Sutherland, 2011). Moreover, different types of valence are associated with different brain regions (Kensinger and Schacter, 2008).
3. The idea that emotional experiences can be assigned to discrete categories such as fear, anger, or happiness could be an obstacle. On the neuronal level, the search for unique signatures of discrete emotions did not produce tangible results. Specifically, co-occurring sets of neuronal features differentiating between such categories were not found (Barrett, 2006). Perhaps it is more expedient to assume more basic psychological processes not being directly associated with emotion, but combined in various ways to produce different emotional and affective states (see Kober et al., 2008).
4. The kind of attentional process that is addressed by the experimental design significantly influences the potential brain areas that came into question to mediate emotion–attention interactions. Consequently, the kind of attentional process should be specified as precisely as possible because of the heterogeneity of labels and understandings as outlined above. With respect to overt and covert attention, for example, Bisley (2011) provided a recent review focusing on associated brain networks.
5. Future research would benefit from the distinction between an internally located vs. externally located emotional impacts on attention. It is important to differentiate between emotional states within subjects and external stimulation with emotion-laden stimuli as demonstrated nicely in a study by Lee and colleagues (2010) participants observed ambiguous visual stimuli that consisted of emotional faces embedded in different levels of visual white noise. Interestingly, when participants thought to look at an emotional face while they actually observed a neutral face, the type of enhanced activity in posterior visual regions was identical with the activity found during the perception of emotional faces.

## The impact of personality traits on attention

Investigations of attentional processes commonly focus on universal mechanisms. Despite ever-present inter-individual variance, the view prevails that attentional processes are comparable between individuals. Given this point of view, sampling from a fictive population of human subjects is an easy step, since it should not significantly matter which subjects constitute the resulting sample. In the end, all values in the dependent variables are averaged across subjects, and an effect of the treatment is eventually observable. However, inter-subject variance may be large, and sometimes subgroups can be identified that differ noticeably from each other but are homogenous within. For example, Pessoa et al. (2005) used a design which leaned on the above-mentioned study by Whalen et al. (1998) who showed that the amygdala responded differentially to masked fearful and happy faces. In contrast, Phillips et al. (2004) did not find any differential response to masked faces in the amygdala, though they also presented emotional target faces for only 30 ms before masking it by a neutral face. Pessoa et al. (2005) showed that no universal objective awareness threshold exists for fear perception. They varied the duration for which a target face was presented before immediately masking it by a neutral face. A substantial percentage of their participants (64%) were able to reliably detect target faces presented for only 33 ms, and some participants even detected fearful faces that were presented for only 17 ms. Obviously, individuals significantly differ in their sensitivity to emotion-laden stimuli, which is perhaps based on individual differences in the general threat sensitivity (Etkin et al., 2004).

The field of differential psychology explicitly addresses such inter-individual differences. However, even here only few studies make an effort to define a real target population with a representative sample. Instead, including many of our own eye-tracking studies, participants are mostly recruited from local educational programs with multiple resulting biases in the sample composition.

Importantly, we do not refer to inter-individual differences induced by context-dependent fluctuations of the physical, mental, or emotional constitution of individuals or plain measurement noise. Rather, we here discuss time- and context-invariant personality traits. Indeed, a whole battery of tests is available to address personality traits in psychological diagnostics. The d2 test, for example, (Brickenkamp, 2002) assesses individual differences in attention ability and identifies attentional deficits. However, the two fields of differential psychology and physiological/psychophysical studies of attention and emotion are largely separated. Only a few studies of visual attention control for inter-individual differences in general attention ability in order to eliminate potential confounding variables (e.g., Hamborg et al., 2012). In the following, we want to illustrate how manifold the impact of time-invariant personality traits on attention can be.

Eizenman et al. (2003) presented slides containing four images. Each of the four images fell into another thematic category: neutral, loss and sadness, threat and anxiety, interpersonal attachment, and social contact. Clinical subjects with depressive disorder and a nonclinical control group observed these slides for an extended period of time. Simultaneous recording of eye movements revealed that the clinical subjects spent more time looking at images depicting loss or sadness than the control group did. Furthermore, mean fixation duration on these images was larger for subjects with depression disorder. Moreover, fixation times of both sample groups correlated with the valence ratings. The authors concluded that individuals with depressive disorder selectively attend to mood-congruent visual material. Consequently, an elaborative processing of mood-incongruent stimuli is prevented when they are simultaneously presented with dysphoric images.

Attention research has also focused on the eye movement performance of patients with schizophrenia. They were found to have abnormal smooth-pursuit eye movements; that is, they were not able to adequately follow a moving object with their eyes. This dysfunction in schizophrenia patients was frequently replicated in samples from all over the world, and it was even shown for first-degree biological relatives of schizophrenia patients (Levy et al., 1993).

Several studies focus on the influence of nonclinical personality traits on humans' visual attention. Friesen and Kingstone (1998) showed that normal subjects asked to detect, localize, or identify a target letter on the left or right of a centrally presented face were faster when the gaze of the face was toward, versus away from, the target. This was the case even though faces' gaze direction had no predictive value with respect to the target location and subjects were told so. Based on this finding, Mathews et al. (2003) investigated whether faces showing fearful expressions enhance the effect of another's gaze in directing the attention of an observer and whether this effect correlates with the trait scale of the State-Trait Anxiety Inventory (STAI) (Spielberger et al., 1993). For that purpose, Mathews and colleagues presented photographs of four men and four women showing either a neutral facial expression or a fearful one. In the original, the eyes of all eight persons looked straight ahead. In two further versions of the photographs, the pupils were moved either to the far left or right corner of both eyes. Each experimental trial started with the original version of a photograph presented in the middle of the screen. It either remained there or was subsequently replaced by the same photograph with eyes shifted left or right. After a varying time interval, one target letter appeared on the left or on the right side of the face, whereby the gaze direction was congruent, neutral, or incongruent to the location of the target letter. Participants had to respond as fast as possible by pressing the corresponding key. Response latencies and errors served as dependent variables and were analyzed by additionally considering inter-individual differences in trait anxiety. Results showed that attention is more likely to be guided by the direction of fearful rather than neutral gaze, but only in anxiety-prone individuals. Overall, faster responses were found in congruent than in incongruent trials, and this congruency effect was similar in both groups with respect to neutral faces. However, when fearful faces were shown, the congruency effect was significantly larger in the group of highly anxious persons in contrast to lowly anxious ones.

It can be concluded that the attention focus of highly anxious individuals is more affected by faces showing fearful facial expressions indicating some danger in the environment, whereas lowly anxious persons do not orientate their attention toward the gaze direction of fearful others. Interestingly, in contrast to the highly anxious group, the lowly anxious group made faster responses to neutral faces in congruent as well as incongruent trials. This result pattern suggests a simple argument: internally located fear hampers fast reaction.

This slowing of disengaging one's attention from a visual cue (i.e., the gaze direction of the faces) shows the complexity of the process. Indeed, a study on the physiology of such disengagement of attention by Khayat et al. (2006) showed that monkeys' attention can be rapidly allocated to newly appearing objects before their attention disengages from a previously attended object.

In a meta-analysis, Bar-Haim et al. (2007) analyzed a total of 172 studies including 2263 anxious and 1768 non-anxious subjects to reveal the boundary conditions of the threat-related attentional bias, that is, a higher sensitivity to threat-related stimuli than to neutral stimuli in highly anxious (but not in lowly anxious) persons. They found across all studies that a significant threat-related bias was present in highly anxious subjects but not in lowly anxious subjects. Moreover, this bias did not depend on the experimental paradigm and was found under varying experimental conditions. Furthermore, the size of the threat-related bias is comparable between anxious children and anxious adults. The mean effect size is *d* = 0.45, indicating a midsize effect (Cohen, 1988).

Perlman et al. (2009) conducted an eye tracking experiment to investigate visual scanpath characteristics evoked by emotional facial expressions. In this context, they especially focused on individual differences in personality. Participants had to freely observe prototypic emotional facial expressions, including happy, angry, fearful, sad, surprised, disgusted, and neutral expressions. No further task was implemented. Before the eye tracking session, subjects had to fill out the Neo Five-Factor Inventory to assess the “big” dimensions of personality, namely extraversion, neuroticism, agreeableness, openness, and conscientiousness. Results revealed that the amount of time spent looking at the eyes of fearful faces was positively related to neuroticism. Hence, and in accordance with the above-described findings of Eizenman et al. (2003), a trait congruency model is supported; that is, individuals seem to search for information that is congruent to their personality traits and avoid incongruent material.

Moreover, Rauthmann and colleagues (2012) also measured the big five personality traits as well as participants' motivation in terms of behavioral inhibition and activation. Participants observed abstract images while their eye movements were recorded. The authors demonstrated with linear mixed models that neuroticism, extraversion, openness, and the behavioral activation system predicted the signature of eye movement parameters, namely the number of fixations, the mean duration of fixations, and the dwelling time within specific areas of interest.

However, at this point we should emphasize that the obvious impact of certain personality traits on attention processes is often more complex and not as obvious as it seems against the background of the above described studies. We know from differential psychology that a certain behavior is always the result of the interplay between personal dispositions such as needs or personality traits and current situational conditions; these can interact in complex ways (Heckhausen and Heckhausen, 2006). Hence, it is probably insufficient to consider only potential main effects of personality traits on attention processes. Rather, it could be fruitful to elucidate the specific interactions between current situational factors and time-invariant personality traits to reveal further interesting effects. In a recent study Kaspar and König (2011a), we investigated changes in eye movement parameters during complex scenes repeatedly presented in a long sequence of stimuli. In this context, we also considered the subjects' global interest in the stimulus set, as well as their general ability to stay within interesting activities without shifting prematurely to alternative activities labeled as the personality trait “action orientation regarding the performance of activities” (AOP; Kuhl, 1994). We expected that this ability only affects viewing behavior when the stimulus material becomes successively more familiar to the observer and that it is related to its interestingness. In fact, we found that the attention focus became more and more locally expressed by several saccade parameters, fixation distributions on the level of single subjects, and an increasing inter-subject variance of fixation distributions across repeated image observations. Importantly, this general tendency was weaker for the group of subjects who rated the image set as interesting, compared to those subjects who were not interested in the images. Moreover, the effects were partly mediated by subjects' ability to stay within interesting activities without shifting prematurely to alternative activities.

Besides the role of personality traits in terms of motivational or behavioral tendencies, demographic variables were also found to be influential factors on attention: Feng et al. (2007) provided evidence for gender differences in spatial selective attention. In their study, they used the useful-field-of-view task to measure spatial cognition. This paradigm allows measuring the ability to detect, localize, and identify a target stimuli as well as the spatial distribution of attentional resources over the field of view. As far as subjects having very little video game experience, men performed better than women. The difference diminished when video game experience was high. Interestingly, in a second study, they were able to show that 10 h of training with an action video game improved performance measurements for spatial attention, whereby women benefited more than men. This implies that balancing men and women in between-subject study designs or considering the gender ratio when interpreting results in within-subject designs is important. Moreover, potential inter-individual differences in domain-specific expertise should also be considered if these potentially influence the dependent variables. In this context, Tanaka and Curran (2001) showed that very specific expertise can change EEG signatures. In their study, dog experts as well as bird experts had to categorize objects. In contrast to categorization of objects outside their domain of expertise, categorization of objects within their respective domains of expertise induced an enhanced early negative component. Consequently, it is mandatory to collect useful metadata, such as demographic variables or domain specific expertise, to appropriately control for these confounding variables. This can be done by adapting the study designs or explicitly introducing inter-individual differences as independent variables in the study design. Also, as suggested by Polyhart and Vandenberg (2010) for the field of longitudinal research, it seems necessary to break out of the “take-what-we-can-get” mentality regarding sample selection in cognitive science.

In line with this suggestion, we discuss that subjects' age also can produce substantial artifacts in the results if it is not considered in sample selection or when assigning subjects to different groups in between-subjects designs.

Mather and Carstensen (2003) examined age differences in attention to and memory for faces. In their experiment, two faces were placed on the left and on the right side of a screen. One of these faces had an emotional expression (sadness, anger, or happiness); the other face was neutral. After both faces disappeared, one dot was shown either on the left or the right position where the faces were previously presented. The dot remained on the display until the participants pressed a corresponding key. Results showed that older adults responded faster to this dot when it was presented on the same side as a neutral face than when it was located on the same side as a negative face. This attentional bias was not found for younger adults. Moreover, older adults remembered positive faces better than faces with negative facial expressions. Mather and Carstensen concluded that older adults obviously avoided negative information and that this attentional bias was consistent with older adults' generally better emotional well-being and their tendency to remember negative less accurately than positive information.

Acik et al. (2010) compared the viewing behavior of three age groups (7- to 9-year-old children, 19- to 27-year-old young adults, and older adults above 72 years) on natural and complex scenes before performing a delayed patch recognition task. Their results suggested that bottom-up mechanisms play a more important role in younger ages: eye movements of young children were heavily guided by basic image features, whereas older adults' viewing behavior was less feature-related. In addition to this differential effect of age on feature-fixation correlations, the explorativeness—that is, the spread of fixation distributions—correlated with feature-related viewing negatively in younger ages. In contrast, older adults increased their feature-related viewing behavior by being more explorative, leading to a better performance in the subsequent patch recognition task. Consequently, it seems that basic image features lose their impact on attention guidance as age increases, and this is paralleled by a stronger impact of top-down processes.

To conclude, we suggest that future studies on humans' attention would benefit from experimental designs that are appropriate for revealing personality influences on viewing behavior. For that purpose, study designs have to be ecologically valid, since very artificial experimental paradigms can cap the impact of personality differences. In this context, a pre-experimental definition of the target population and a subsequent sampling of an appropriate (i.e., representative) sample should be considered.

### Conflict of interest statement

The authors declare that the research was conducted in the absence of any commercial or financial relationships that could be construed as a potential conflict of interest.
